# Identification of a Cancer Stem Cell-Related Gene Signature in Hepatocellular Carcinoma Based on Single-Cell RNA-Seq and Bulk RNA-Seq Analysis

**DOI:** 10.3390/ijms26072933

**Published:** 2025-03-24

**Authors:** Jing Wu, Xu Liu, Sheng Huang, Wei Liu

**Affiliations:** 1 Medical School, Hubei Minzu University, Enshi 445000, China; liuxu594666@gmail.com (X.L.); 2015026@hbmzu.edu.cn (S.H.); 2 Department of Bioinformatics and Computational Biology, The University of Texas MD Anderson Cancer Center, Houston, TX 77030, USA

**Keywords:** Cancer Stem Cells (CSCs), Liver Hepatocellular Carcinoma (LIHC), prognosis, biomarkers, immune infiltration

## Abstract

Cancer stem cells (CSCs) are a heterogeneous group of tumor cells that play a significant role in tumorigenesis, therapeutic resistance, and recurrence in liver hepatocellular carcinoma (LIHC). This study combines clinical data sets from The Cancer Genome Atlas (TCGA) and the International Cancer Genome Consortium (ICGC) with bulk RNA sequencing data. This study also features the GSE156625 single-cell RNA sequencing (scRNA) data set from the GEO to explore the prognostic significance of CSC biomarkers (BCSCs) in LIHC. In this research, we introduce a developed prognostic risk model that relies on nine specific BCSCs, including ADM, CCL5, CD274, DLGAP5, HOXD9, IGF1, S100A9, SOCS2, and TNFRSF11B. It was found that high-risk patients experience shorter overall survival rates when compared to low-risk patients. Additionally, the study characterized the composition of immune cells within the tumor microenvironment (TME) and revealed significant variations in gene-expression levels and mutation rates between different risk groups. The model suggests that liver cancer progression might be driven by immune evasion independent of PD-L1 and highlights the potential of the low-risk BCSC group being sensitive to various treatments. Our findings offer a promising foundation for personalized LIHC therapy and highlight the need for further experimental validation of the roles of these CSCs in disease progression.

## 1. Introduction

Hepatocellular carcinoma (HCC) represents a serious threat to global health, ranking as the sixth most commonly diagnosed cancer and the third primary contributor to cancer-associated fatalities worldwide, based on GLOBOCAN 2022 data [1]. Despite recent therapeutic advancements in HCC, including the adoption of chemotherapy, surgical interventions, and immunotherapy, the global mortality of HCC has persistently increased [2]. High mortality and recurrence rates in HCC are partly due to liver cancer stem cells (CSCs).

Recent findings show that CSCs have stem cell characteristics that are crucial for liver cancer metastasis, recurrence, and therapeutic resistance [3,4,5]. The cancer stem cell model suggests that tumor growth is driven by a specific group of stem cells within the tumor [6]. However, differentiating CSCs from the larger cancer cell population remains a formidable challenge [7]. Previous studies have used specific markers to differentiate liver CSCs, including CD133, CD90, CD44, OV6 (an oval cell marker), EpCAM, CD13, CD24, DLK1, and CD47 [8]. Recent studies have shown that BCSC-positive cells in HCC patients are linked to poor prognostic outcomes, suggesting their potential in forecasting the disease’s trajectory. Kong et al. revealed that elevated PD-L1 levels in CD133+ liver CSCs and CSC-enriched tumor spheres activate the SGK2/β-catenin pathway, thereby causing epithelial-mesenchymal transition (EMT) and the development of stem cell characteristics [9]. Patients with positive markers for EpCAM, CD90, CD44, or CD133 in HCC often experience worse prognostic outcomes. These include more advanced tumor-node-metastasis (TNM) stages, increased tumor infiltration, metastasis, and greater drug resistance [10]. Specifically, the CD133+, CD44+, or CD133+/EpCAM+ liver CSCs, have been shown to exhibit resistance to sorafenib [10].

Tumor-associated macrophages (TAMs) are involved with CXCL1 and CXCL2. They also influence the downstream CXCR2/ERK signaling pathway. This indicates that they may serve as potential therapeutic targets to combat sorafenib resistance in HCC [11]. CSC markers have been shown to correlate with the immunosuppressive tumor microenvironment (TME). This correlation offers valuable insights for immunotherapy in HCC patients [12]. However, some studies have explored the functional attributes of specific CSC markers; there has been no comprehensive analysis of their roles in HCC treatment. Using data from TCGA–LIHC and ICGC, we built and confirmed a nine-gene risk-prediction model for HCC to fill this gap.

Given the critical role of CSCs in promoting chemoresistance and facilitating tumor recurrence, a deeper understanding of CSC signatures could significantly refine the selection criteria for therapeutic interventions [13]. Recent studies have demonstrated that BCSCs can serve as predictors of treatment response in HCC [14]. Thus, reducing the population of BCSCs is crucial for improving therapy effectiveness in advanced HCC [10,15]. Additionally, ongoing research suggests that CSC-associated pathways are promising targets for immunotherapy, potentially improving treatment outcomes in tumors typically resistant to immune-based therapies [16]. Incorporating CSC profiling into clinical practice could lead to more personalized treatment plans, ultimately enhancing patient outcomes by tailoring therapies to the unique tumor biology of each patient.

This study shows that CSC diagnostic markers can help categorize HCC patients, providing insights into predicting outcomes and customizing treatment strategies. The crucial role of CSCs in cancer progression highlights the importance of investigating their associated genes, which could lead to promising treatment pathways.

## 2. Results

### 2.1. Identification of LIHC–Specific BCSC–Related Genes and Functional Analysis

**Figure 1:**
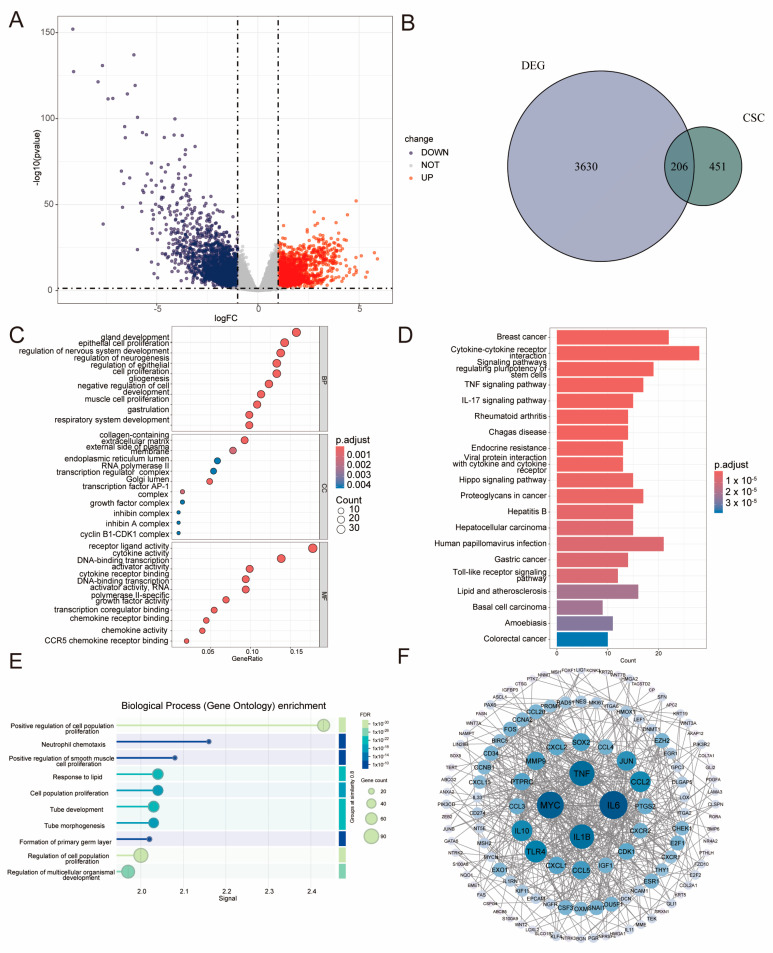
Identifying differentially expressed biomarkers of cancer stem cells (DE–BCSCs) in the TCGA–LIHC cohort. (**A**) Volcano plot illustrates the differentially expressed genes (DEGs) in LIHC, using red for significantly upregulated (UP) genes, blue for downregulated (DOWN) genes, and gray for non-significant (NOT) genes. (**B**) Venn diagram of the 206 overlap genes between DEGs and BCSCs. (**C**) In the dot plot, the 10 most enriched gene ontology terms are displayed, categorized into biological processes (BP), cellular components (CC), and molecular functions (MF). (**D**) A bar chart illustrates the 20 most significantly enriched signaling pathways categorized by KEGG classifications. (**E**) A lollipop chart illustrates the biological pathways of the 206 overlapping genes in the STRING database. (**F**) Protein–protein interaction (PPI) network of 131 core DE–BCSCs.

After data preparation, 3,630 differentially expressed genes (DEGs) were identified from 401 LIHC samples (Figure 1A). Using established criteria, we identified 206 differentially expressed biomarkers of cancer stem cells (DE–BCSCs) (Figure 1B) that indicate prognostic significance. Among the identified genes, 93 were found to be upregulated and 113 downregulated in HCC. Gene Ontology (GO) and KEGG functional analysis revealed that the DE–BCSCs play a crucial role in both cell proliferation and differentiation processes. Specifically, these processes include epithelial cell proliferation, gland development, and nervous system development (Figure 1C). KEGG pathway analysis further demonstrated that the DE–BCSCs play pivotal roles in various cancer-related pathways, including breast cancer, hepatocellular carcinoma, gastric cancer, pathways regulating the pluripotency of stem cells, cytokine–cytokine receptor interaction, and the TNF and IL-17 signaling pathways (Figure 1D). Additionally, the protein-protein interaction (PPI) network analysis identified strong interactions among 131 genes within the network. In the context of DE–BCSCs, these findings highlight the significance of the positive regulation of the cell population proliferation pathway (Figure 1E,F).

### 2.2. Construction of a Prognostic Model Using DE-BCSCs

**Figure 2:**
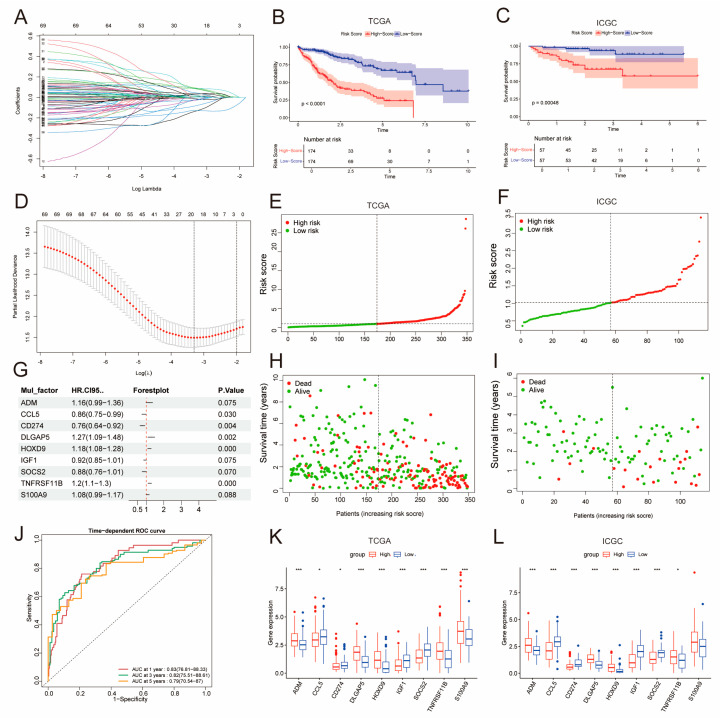
Developing and validating a prognostic model for liver cancer. (**A**,**D**) Lasso–Cox regression analysis of DE–BCSCs to identify key signature genes. including coefficient profiles of genes across log-transformed Lambda values (**A**) and 10-fold cross-validation to identify the optimal Lambda (**D**). (**B**,**C**) The Kaplan–Meier survival analysis was conducted for HCC patients, categorizing them into high-risk and low-risk groups according to the median risk signature derived from the TCGA–LIHC training cohort (**B**) as well as the ICGC validation cohort (**C**). (**E**,**F**) Risk score curve depicting the categorization of patients into low-risk (green) and high-risk (red) groups. (**G**) Multivariate Cox regression analysis identifying BCSCs associated with patient prognosis. (**H**,**I**) The risk score scatter plot shows red dots representing deceased patients and green dots representing survivors; a higher risk score correlates with increased mortality. (**J**) Survival-dependent ROC curves illustrating the OS rates at 1, 3, and 5 years for patients in TCGA–LIHC are presented in TCGA–LIHC patients. (**K**,**L**) A comparison of the expression profiles of nine diagnostic genes between two risk groups within TCGA–LIHC (**K**) and ICGC cohorts (**L**), * for *p* ≤ 0.05 and *** for *p* ≤ 0.001.

**Figure 3:**
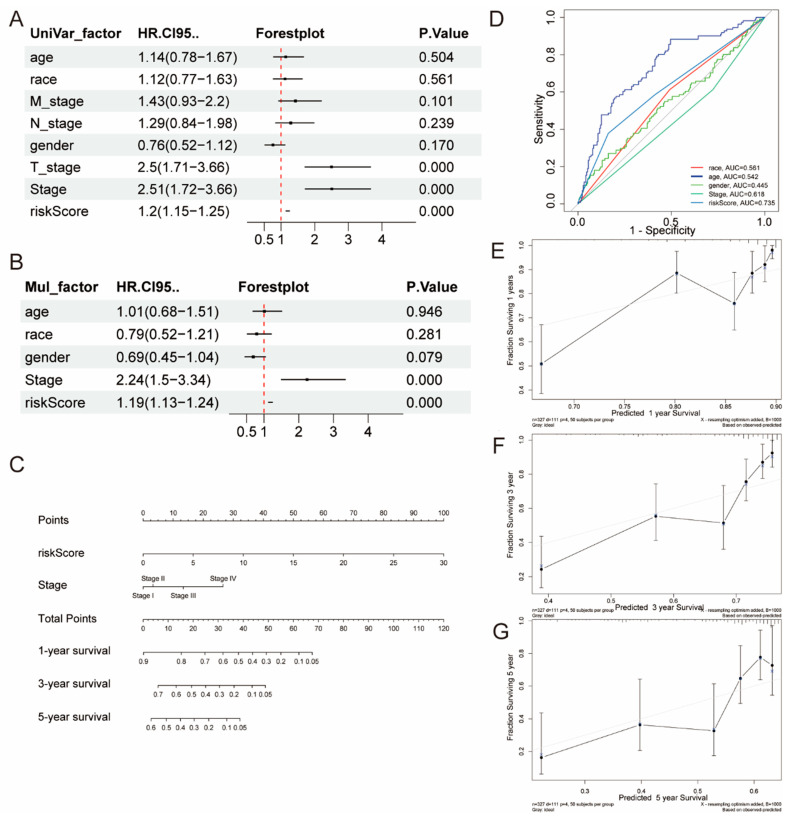
The creation and evaluation of a nomogram model. Both univariate (**A**) and multivariate (**B**) Cox proportional hazards regression analyses were performed to evaluate clinical characteristics and selected clinical characteristics, respectively. (**C**) Nomogram model predicting 1-, 3-, and 5-year survival probabilities for TCGA–LIHC patients. (**D**) ROC curves for clinical features and risk scores. (**E**–**G**) Calibration curves for the nomogram predictions of 1-year (**E**), 3-year (**F**), and 5-year (**G**) overall survival (OS) in the LIHC cohort.

We carried out a basic Cox regression analysis with one variable to evaluate how well the 206 DE–BCSCs can differentiate LIHC patients from control subjects. The analysis identified 26 genes that reduce the risk of the disease, indicated by hazard ratios (HR) less than 1, and 45 genes that increase the risk, indicated by HR greater than 1 (Figure S1). To evaluate their potential as prognostic indicators, we first performed LASSO analysis, then conducted multivariate Cox proportional hazards regression analysis on these genes (Figure 2A,D). Finally, nine prognostic genes were identified: ADM, CCL5, CD274, DLGAP5, HOXD9, IGF1, SOCS2, TNFRSF11B, and S100A9 (Figure 3G). Following this, we derived a computational equation to represent the prognostic risk score. BCSC score = 0.145 × ADM expression − 0.148 × CCL5 expression − 0.268 × CD274 expression + 0.241 × DLGAP5 expression + 0.163 × HOXD9 expression − 0.079 × IGF1 expression − 0.133 × SOCS2 expression + 0.180 × TNFRSF11B expression + 0.075 × S100A9 expression.

Risk scores were determined for two distinct cohorts: TCGA–LIHC and ICGC. The scores, which were obtained from the expression levels of prognostic genes along with their respective regression coefficients, served to categorize patients into high-risk and low-risk categories according to the median risk score. Survival analysis revealed distinct outcomes between the groups. Specifically, the group identified as low-risk exhibited superior survival rates in all cohorts, as evidenced by a log-rank test *p*-value of less than 0.0001 for the TCGA data set (see Figure 2B) and a *p*-value of 0.00048 for ICGC (see Figure 2C). A total of nine diagnostic genes exhibited significant differential expression when comparing the high-risk group to the low-risk group (Figure 2K,L), and to enhance understanding, we generated time-dependent receiver operating characteristic (ROC) curves for 1-year, 3-year, and 5-year intervals for each data set. Significantly, the Area Under the Curve (AUC) for the 3-year time-dependent ROC analysis was greater than 0.82 across the LIHC cohort, indicating strong predictive capabilities of the prognostic risk score (Figure 2J). Additionally, risk plots illustrated the distribution of risk scores (Figure 2E,F) across all data sets. They also showed the associated overall survival (OS) status (Figure 2H,I). A key finding was the clear link between higher prognostic risk scores and an increase in patient mortality.

### 2.3. The Creation and Verification of a Nomogram Model

We conducted both univariate and multivariate Cox regression analyses utilizing the TCGA data set, concentrating on clinical variables and risk scores to identify essential prognostic factors. Our results demonstrated a significant association between both the risk score and tumor stage with the prognosis of LIHC patients (*p* < 0.05), highlighting their importance in clinical assessments (Figure 3A,B). We used AUC curves to evaluate the predictive capability of the risk score and compare it with clinical factors such as race, age, grade, gender, and stage. The risk score achieved the highest AUC value of 0.735 (Figure 3D).

The results of the survival analysis indicated that individuals in the low-risk category had prolonged OS compared to those in the high-risk category. Using these factors, we developed a nomogram aimed at forecasting the survival probabilities for individual patients at 1 year, 3 years, and 5 years (Figure 3C). To further validate the model, we developed calibration curves for the predictions spanning 1 year, 3 years, and 5 years to assess the efficacy of the nomogram in differentiating among various survival outcomes (Figure 3E–G).

### 2.4. Identification of DEGs and Gene Set Enrichment Analysis (GSEA)

**Figure 4:**
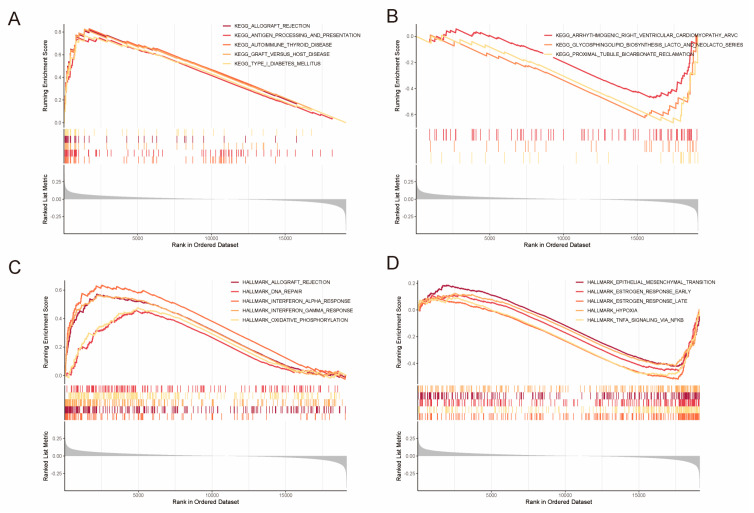
Gene Set Enrichment Analysis of DEGs and somatic mutation characteristics between two risk groups. (**A**,**B**) The GSEA outcomes reveal the presence of enriched gene sets within the C2 collection, specifically highlighting the KEGG pathways associated with both the high-risk cohort (**A**) and the low-risk cohort (**B**). Each distinct gene set is represented by a unique colored line, illustrating the upregulated genes positioned to the left (adjacent to the origin) and the downregulated genes located on the right side of the *x*-axis. This visualization includes only the most significant gene sets. (**C**,**D**) Enriched gene sets from the HALLMARK collection for the high-risk cohort (**C**) and low-risk cohort (**D**).

Our objective was to gain a deeper insight into the functional consequences of the nine BCSC gene signatures in LIHC. To achieve this, we conducted GSEA with TCGA–LIHC gene-expression data, stratifying patients into low- and high-risk groups. In the high-risk group, the five KEGG pathways that showed significant enrichment were type 1 diabetes mellitus, allograft rejection, asthma, graft-versus-host disease, and autoimmune thyroid disease (Figure 4A). In contrast, the most prevalent KEGG pathways in the low-risk group included proximal tubule bicarbonate reclamation, glycosphingolipid biosynthesis (lacto and neolacto series), and arrhythmogenic right ventricular cardiomyopathy (ARVC) (Figure 4B). Additionally, hallmark pathways such as DNA repair, oxidative phosphorylation, interferon-gamma response, allograft rejection, and interferon alpha response were significantly enriched in the high-risk group (Figure 4C). In the low-risk group, there was significant enrichment of estrogen response (late and early), TNF-alpha signaling via NF-kB, epithelial-mesenchymal transition, and hypoxia (Figure 4D).

### 2.5. Elucidation of DE–BCSCs as Potential Protective Factors

**Figure 5:**
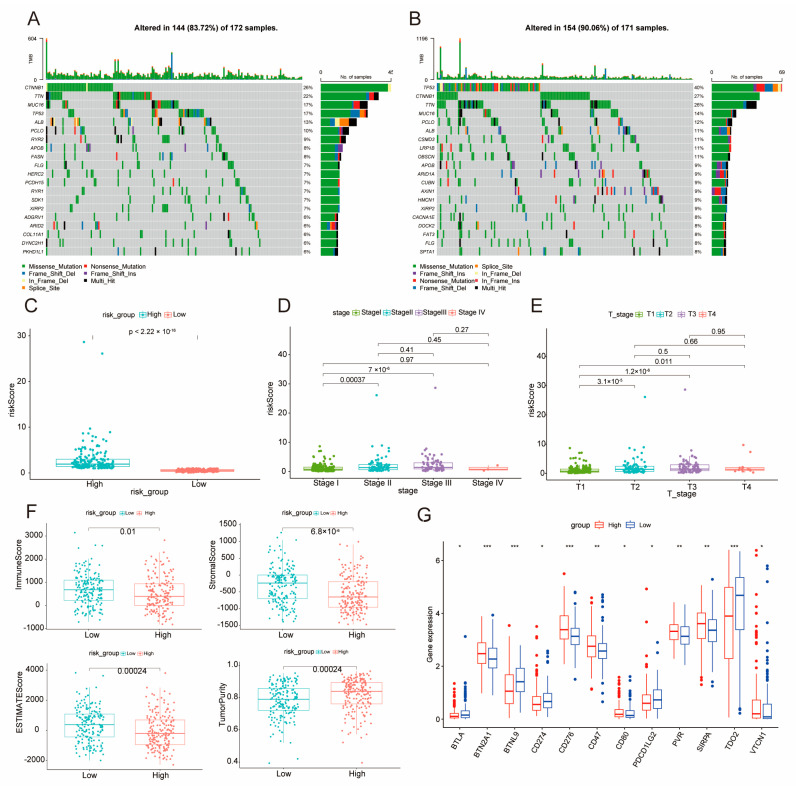
Genetic Alterations and Risk Scores Across Clinical Characteristics in LIHC Cohort. (**A**,**B**) Oncoplots of genetic alterations in the low-risk group (**E**) and high-risk group (**F**), showing the types and frequencies of mutations in top mutated genes. (**C**–**E**) Comparison of risk scores across clinical parameters: risk groups (high vs. low) (**C**), clinical stages (Stage I–IV) (**D**), and T-stages (T1−T4) (**E**); (**F**) Differences in immune score, stromal score, ESTIMATE score, tumor purity between the high and low-score groups. (**G**) Expression analysis of immune checkpoint genes between the two risk groups, * for *p* ≤ 0.05, ** for *p* ≤ 0.01 and *** for *p* ≤ 0.001.

We investigated genetic alterations by analyzing and visualizing the mutation profiles of low-risk (Figure 5A) and high-risk (Figure 5B) HCC patient groups using oncoplots. The occurrence of mutations within the high-risk subgroup was found to be 90.06%, which is notably greater than the 83.72% observed in the low-risk subgroup; both groups exhibited several frequently mutated genes, including TP53, CTNNB1, TTN, MUC16, ALB, PCLO, APOB, and MUC4. Both subgroups shared several frequently mutated genes, including TP53, CTNNB1, TTN, MUC16, ALB, PCLO, APOB, and MUC4. In the LIHC cohort, we evaluated risk scores based on various clinical characteristics (Figure 5C). We observed a progressive increase in risk scores across clinical stages, from Stage I to IV (Figure 5D). Similarly, risk scores exhibited significant variation across T−stages (T1−T4 and later stages vs. early stages) (Figure 5E and Figure S5). Further analysis identified notable discrepancies in TME metrics when comparing high-risk and low-risk groups.

The analyzed metrics, which included immune scores, stromal scores, ESTIMATE scores, and tumor purity (Figure 5F), all had *p*-values less than 0.05. Moreover, immune checkpoint gene-expression levels differed significantly between the two identified risk groups. (Figure 5G, *p* < 0.05). These findings highlight the distinct genetic and clinical features associated with risk scores and suggest that these features may influence the TME and immune modulation in LIHC.

### 2.6. The Distribution of Tumor-Infiltrating Immune Cells (TICs) Across Two Distinct Subgroups

Immune cell infiltration is instrumental in influencing drug resistance, tumor progression, and patient prognosis. Given the heterogeneity of HCC, the intricate interplay between immune cells and cancer stem cells are fundamental in determining survival outcomes in liver cancer patients.

**Figure 6:**
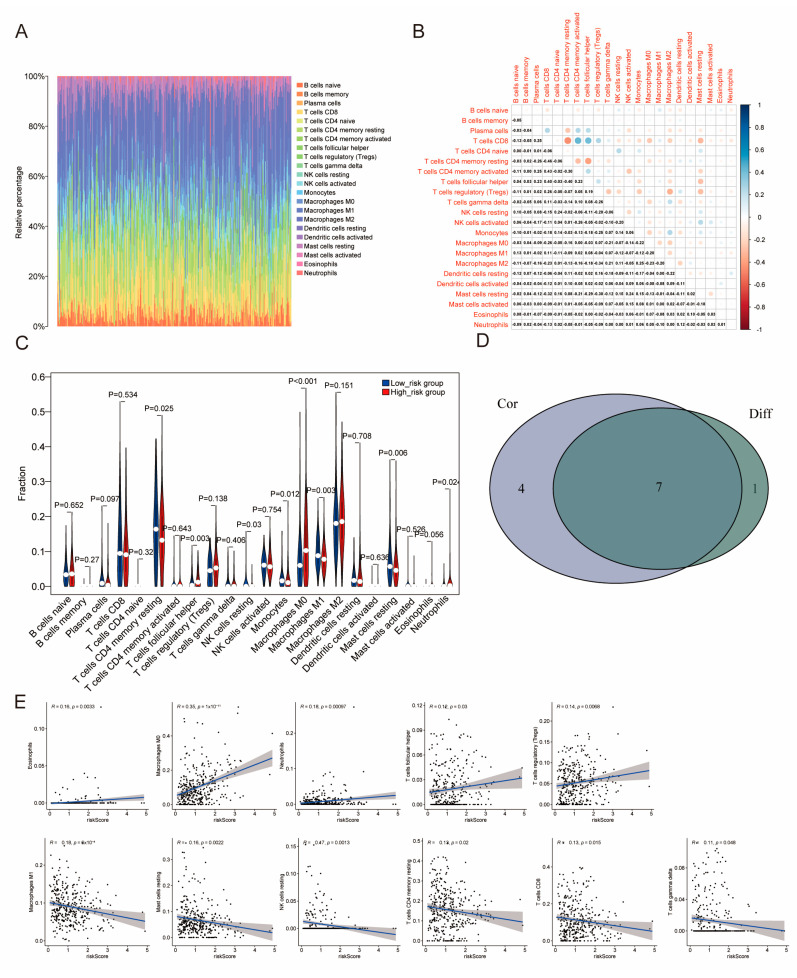
The tumor-infiltrating cell (TIC) profile in LIHC samples and correlation analysis between risk score and immune cell infiltration. (**A**) A bar graph illustrating the proportions of 22 TIC types in LIHC tumor samples, with columns labeled by sample ID. (**B**) Heatmap illustrating correlations among 22 immune cell types. Each box shows the *p*-value for the correlation between two cell types, with shading representing the correlation strength. (**C**) Violin plot comparing immune cell ratios between two risk groups in LIHC tumor samples. The significance of the results was assessed using the Wilcoxon signed-rank test. (**D**) Venn diagram showing seven TIC types associated with risk score, identified by both differential and correlation analyses in violin and scatter plots, respectively. (**E**) Scatter plots showing correlations between the proportions of 11 TIC types and risk scores (*p* < 0.05). Each plot includes a fitted linear model (blue line) to illustrate the trend of immune cell proportions with a risk score, with correlation assessed using the Pearson coefficient.

To explore this relationship, the CIBERSORT algorithm was employed to analyze the distribution of 22 types of TIC across LIHC tumor samples (Figure 6A). A heatmap was generated to illustrate the correlations among these TIC types, with shading reflecting the strength and direction of the correlations (Figure 6B). A violin plot demonstrated notable distinctions in the proportions of immune cell types between two risk groups (*p* < 0.05) (Figure 6C). The subsequent analysis identified seven TIC types that were significantly linked to risk scores through differential and correlation analyses. Comparative analysis showed distinct patterns of immune cell infiltration across the various risk groups. The high-risk cohort exhibited markedly higher percentages of resting CD4 memory T-cells, activated CD4 memory T-cells, follicular helper T-cells, M0 macrophages, and neutrophils.

In contrast, the low-risk cohort had a higher number of resting NK cells, monocytes, M1 macrophages, and resting mast cells. This variation in immune cell distribution suggests potential biological mechanisms that influence risk stratification. Analysis identified seven TIC types significantly associated with risk scores through both differential and correlation analyses. A comparative analysis showed varying patterns of immune cell infiltration among the risk groups. The high-risk cohort contained significantly higher proportions of resting CD4 memory T-cells, follicular helper T-cells, M0 macrophages, and neutrophils. In contrast, the low-risk cohort had more resting NK cells, monocytes, M1 macrophages, and resting mast cells. This variation in immune cell distribution suggests potential biological mechanisms underlying risk stratification. (Figure 6D). Scatter plots revealed significant correlations between the proportions of 11 TIC types and risk scores (*p* < 0.05). Fitted linear models indicated trends in immune cell proportions as risk scores increased, with correlation strength quantified using Pearson coefficients (Figure 6E). Notably, five TIC types, including Eosinophils, M0 macrophages, Neutrophils, follicular helper T-cells, and regulatory T-cells (Tregs), demonstrated a favorable association with the risk signature. Conversely, six TIC types—M1 Macrophages, resting Mast cells, resting NK cells, resting CD4 memory T-cells, CD8 T-cells, and gamma delta T-cells—showed a negative association with the risk signature. These findings underscore the significant associations between TIC profiles and risk scores, offering new insights into the immune landscape and its potential role in risk stratification and disease progression in LIHC.

### 2.7. The Single-Cell Analysis Predicts the Expression Distribution of Model Genes and Intercellular Communication

We conducted a scRNA-seq analysis to characterize the immune cell composition and the geneexpression patterns present in the TME of HCC patients.

**Figure 7:**
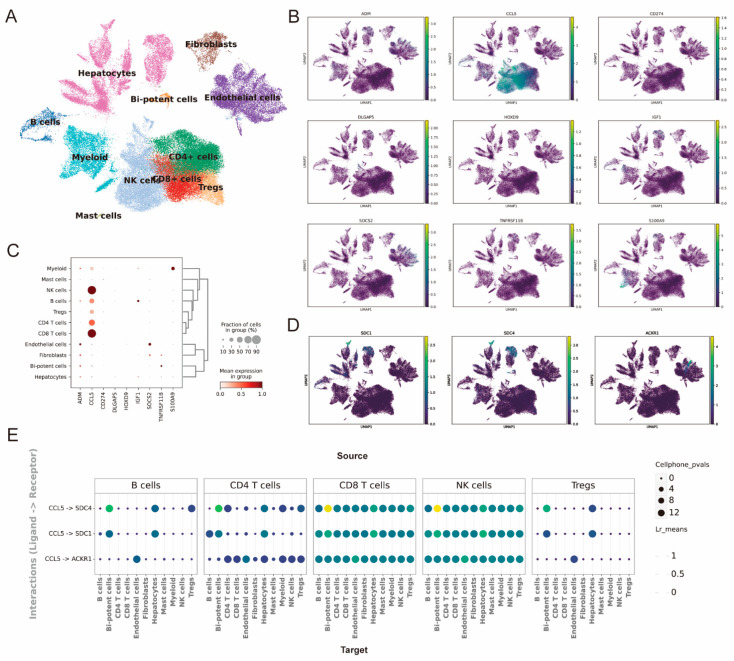
Immune cell composition and gene-expression analysis in the tumor microenvironment of HCC. (**A**) UMAP visualization of immune and non-immune cell populations in the TME of HCC samples. (**B**) UMAP plots showing the expression of specific genes (ADM, CCL5, CD274, DLGAP5, HOXD9, IGF1, SOCS2, TNFRSF11B, and S100A9) across the identified cell populations, mapped using the viridis palette from low (dark blue-purple) to high (yellow). (**C**) Dot plot showing the cell fraction and mean gene expression across different cell types. Dot size corresponds to the percentage of cells in each group expressing the gene, while color intensity (light red to dark red) represents the average expression level among expressing cells. (**D**) UMAP visualization of CCL5 target gene-expression patterns (SDC1, SDC4, and ADO2). (**E**) Ligand-receptor interaction analysis using CellPhone DB showing interactions between B-cells, CD4+ T-cells, CD8+ T-cells, NK cells, Tregs, and other immune and non-immune cell populations in the TME. Cell interactions are presented with significance values and mean expression levels mapped using the viridis palette from low (dark blue-purple) to high (yellow).

Uniform Manifold Approximation and Projection (UMAP) clustering revealed that the TME encompasses various cell types. These include hepatocytes, fibroblasts, bipotent cells, endothelial cells, and several immune cells: B cells, myeloid cells, CD4+ T-cells, natural killer (NK) cells, regulatory T-cells (Tregs), and mast cells. The distinct separation of these immune and non-immune cell clusters highlights the heterogeneity of the TME (Figure 7A). Gene-expression analysis of key model genes, such as ADH1, CCL5, CD274, DLSGPS, HOXD9, ISG15, TNFSF11, S100A9, SDC3, SDC4, and ADO2, demonstrated variable expression patterns across different cell clusters (Figure 7B,D). Notably, the percentage of cells that exhibited CCL5 expression was markedly elevated within the clusters comprising B-cells, NK cells, Tregs, as well as CD4+ and CD8+ T-cells (Figure 7C). To examine intercellular communication, we conducted an analysis of ligand-receptor interactions using CellPhoneDB. This identified the top 20 ligand-receptor pairs with the highest interaction intensities between immune cells in the TME and liver cancer cells, including bipotent cells, hepatocytes, and fibroblasts (Figure S3). Notably, distinct expression patterns of pairs like CCL5–SDC1, CCL5–SDC4, and CCL5–ACKR1 were observed across clusters of immune cells encompass various types, including B-cells, CD4+ T-cells, CD8+ T-cells, NK cells, and Tregs (Figure 7E). This research highlights how complex cell-cell communication within the TME may contribute to the progression of HCC. It offers crucial insights into gene expression and cellular interactions, providing a foundation for identifying potential therapeutic targets.

### 2.8. Sensitivity Analysis of Potential Clinical Drugs Within Groups Identified as High-Risk and Low-Risk

**Figure 8:**
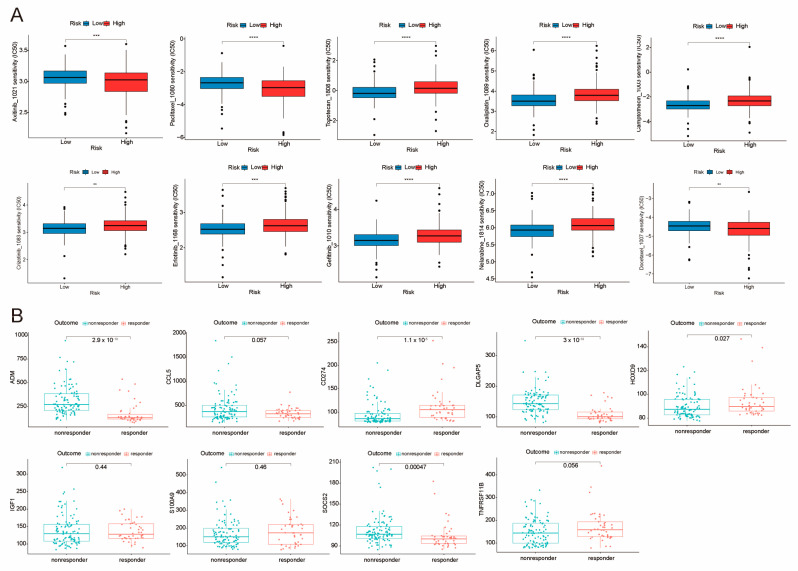
Drug identification from GDSC2 database and Drug Response in HCC patients. (**A**) A comparative analysis of drug sensitivity, measured in terms of IC50 values, was conducted between two risk groups, ** for *p* ≤ 0.01, *** for *p* ≤ 0.001 and **** for *p* ≤ 0.0001. (**B**) Comparative analysis of post-treatment response groups in LIHC patients from the GSE109211 data set. Patients exhibiting diminished expression levels of ADM, CCL5, DLGAP5, and SOCS2, and high expression of CD274 and HOXD9, demonstrated favorable treatment outcomes.

We assessed the drug sensitivity of 10 potential drugs in LIHC patients by comparing their IC50 values, and our findings revealed that the high-risk cohort demonstrated a greater responsiveness to Axitinib and Paclitaxel in comparison to the low-risk cohort. Interestingly, patients with low-risk hepatocellular carcinoma (HCC) displayed an elevated sensitivity to a variety of pharmacological agents, including Topotecan, Oxaliplatin, Camptothecin, Crizotinib, Erlotinib, Gefitinib, Nelarabine, and Docetaxel (Figure 8A). In order to assess the therapeutic effectiveness of the nine identified genes, we analyzed the GSE109211 data set, which includes therapeutic outcome data for LIHC patients. Patients treated with sorafenib showed no significant differences in the expression levels of these nine genes compared to those receiving a placebo treatment (Figure S2). Further analysis indicated that patients with lower expression levels of ADM, CCL5, DLGAP5, and SOCS2, along with higher levels of CD274 and HOXD9, were associated with better treatment outcomes (Figure 8B). Our observations indicated that multiple genes critically involved in the regulation of immune checkpoints, including BTLA, CD276, CD47, PVR, SIRPA, and VTCN1, were found to be expressed at elevated levels within the high-risk cohort, while BTN2A1, BTNL9, CD274, CD80, PDCD1LG2, and TDO2 showed higher expression in the low-risk group. These findings may provide valuable insights for immunotherapy (Figure 5G). These results provide valuable references for the clinical application of immunotherapy in combination with chemotherapy.

## 3. Discussion

HCC is a diverse disease found worldwide, and its prognosis often worsens due to metastatic progression. Currently, a phase-adjusted treatment strategy is adopted for managing recurrent liver cancer [17]. Stratification of HCC is crucial for guiding therapeutic interventions [18]. Recent studies suggest that the CSC model explains various adverse clinical outcomes, including frequent recurrence after therapy, tumor dormancy, and treatment resistance [5]. Nevertheless, the precise molecular mechanisms by CSCs that contribute to the advancement of HCC are still not well-understood. Our study resulted in a prognostic signature derived from nine CSC-related genes: ADM, CCL5, CD274, DLGAP5, HOXD9, IGF1, SOCS2, TNFRSF11B, and S100A9. This signature effectively stratifies HCC patients into two distinct risk subgroups. Risk scores were utilized to evaluate differences in survival outcomes, tumor stage, TME, somatic mutation profiles, and drug sensitivity among these subgroups. Our findings offer a more precise prognostic framework for HCC patients, contributing to personalized treatment strategies.

The liver microenvironment helps maintain immune tolerance to prevent abnormal responses to antigens from the portal vein. Cancer immunotherapy boosts tumor-specific immunity by enhancing the anti-tumor functions of effector cells, like cytotoxic cells that kill tumor cells, or by reactivating suppressive TMEs [19]. The TME is essential for CSC development. It promotes their growth and differentiation, which in turn drives tumor progression [20]. Many studies demonstrate that CSCs secrete factors that maintain their stemness and promote angiogenesis, while also recruiting immune cells like macrophages, dendritic cells, and T-cells. Other stromal cells join in by releasing additional molecules that drive tumor progression and contribute to chemotherapy resistance [21]. CSCs secrete immunosuppressive cytokines. These cytokines allow tumor-associated macrophages (TAMs) to release inflammatory cytokines. In turn, these cytokines recruit myeloid-derived suppressor cells (MDSCs) and facilitate the development of a tumor-promoting microenvironment [22]. The findings of this study indicated that there was a greater prevalence of M1 macrophages in the low-risk cohort, in contrast to a higher occurrence of M0 macrophages within the high-risk cohort (Figure 6C). Tumors contain more macrophages than surrounding non-tumor tissues [23], and M1 macrophages are known for their anti-tumor properties [24], as supported by prior research. The proportions of different immune cell types, including M1 macrophages, resting NK cells, and resting mast cells, are linked to the outcome of HCC [25]. Higher levels of these cells are observed in the low-risk group. Furthermore, the higher proportion of NK cells in the low-risk group indicates their involvement in immune surveillance of HCC [26]. Within the high-risk cohort, the percentage of neutrophils and follicular helper T-cells was elevated. This is likely associated with their cytokine secretion that promotes immune evasion and tumor recurrence [27,28].

In immunotherapy, specific immune checkpoint inhibitors (ICIs), including PD-L1 (CD274), serve as significant therapeutic targets. In this study, we observed that CD274 and PDCD1LG2 (PD-L2) had lower RNA expression in tumor specimens and high-risk cohorts when contrasted with normal samples and low-risk groups (Figure 5G), suggesting non-PD-L1-dependent immune evasion mechanisms, such as alternative checkpoint molecules or immunosuppressive cells. Further analysis revealed that immune checkpoint genes CD47, PVR, SIRPA, and VTCN1 were found to be elevated in the high-risk cohort, indicating their role in suppressing anti-tumor immunity (Figure 5G). Increased levels of CD47 prevent macrophages from clearing tumors, while SIRPα binding to CD47 reduces macrophage phagocytosis [29]. VTCN1 (B7-H4) is an immunosuppressive molecule that inhibits T-cell activation and proliferation [30]. Additionally, PVR, through its interaction with TIGIT, suppresses the function of T-cells and NK cells, thereby promoting immune evasion [31]. Additionally, CSC markers ADM, DLGAP5, S100A9, and TNFRSF11B showed consistent overexpression at both RNA and protein levels in tumor samples, with significantly lower expression in normal tissues. In contrast, IGF1, SOCS2, and CCL5 showed higher expression in normal samples (Figure 2K,L). SOCS2 is integral to regulating macrophage infiltration and cellular proliferation, thereby influencing disease progression and its associated severity [32]. TNF Receptor Superfamily Member 11B (TNFRSF11B) is an anti-apoptotic protein that interacts with and inhibits the TNF-related apoptosis-inducing ligand (TRAIL). Suppressing TRAIL may allow TNFRSF11B to reduce HCC spread, potentially improving the prognosis for affected patients [33]. The high-risk group showed greater sensitivity to Axitinib and Paclitaxel, while the low-risk group responded better to various other agents, highlighting distinct therapeutic vulnerabilities in HCC patients (Figure 8A). Notably, genes associated with the CSC model showed significant expression differences in response to Sorafenib, indicating their role in influencing treatment efficacy (Figure 8B). GSEA revealed that the differential genes between risk groups are mainly enriched in pathways associated with chronic inflammation, immune evasion, and immune imbalance. This finding underscores the complexity of the HCC immune microenvironment (Figure 6). These findings highlight alternative immune-evasion pathways and CSC biomarkers as potential therapeutic targets in high-risk HCC.

The model also highlights significant differences in gene function and immune infiltration among various risk categories, which could improve early diagnosis and enable more effective targeted therapies for HCC. However, this study has limitations; primarily, the exact roles of these genes in HCC are not yet clear. Additionally, our data comes from publicly available data sets on HCC patients, which limits its scope. Given the prognostic potential of these markers, further validation through prospective studies is necessary. The diagnostic model using these nine genes effectively distinguishes variations in immune cell compositions among patients and shows promise for predicting tumor-related immune dynamics. It remains crucial to assess this model’s predictive prowess in real-world clinical settings, especially for patients under diverse immunotherapy regimens.

## 4. Materials and Methods

### 4.1. Diagram of Study Flow and Data Collection

We used TCGA biolinks to acquire RNA-Seq data from 357 LIHC samples and 44 control samples in the TCGA database (http://portal.gdc.cancer.gov, accessed on 1 May 2024) [34]. The University of California at Santa Cruz (UCSC) also provided relevant clinical data on LIHC. For this study, we downloaded the GSE109211 [35] and GSE156625 [36] single-cell RNAseq (scRNA) data set from the GEO database (https://www.ncbi.nlm.nih.gov/geo, accessed on 1 May 2024) [37]. Additionally, we obtained a data set of 113 HCC samples [38] from the ICGC database [39]. Our analysis included only HCC samples that had both expression profile data and corresponding clinical details. Additionally, according to the report by Firdous et al., we identified 838 definitive biomarkers. These biomarkers, sourced from the BCSCdb, are listed in Supplementary Table S1 of the database article. These biomarkers are categorized based on a threshold value of ≥0.2, comprising 128 miRNAs and 710 genes [40]. Of these, approximately 657 genes are identified in the RNA–SEQ data sets of LIHC. The research process is illustrated in Figure S4.

### 4.2. Identification of DE–BCSCs in LIHC

We first extracted BCSC expression data from TCGA–LIHC and then identified DE–BCSCs between the LIHC cohort and non-tumor controls using the limma package [41]. We visualized the variably expressed BCSCs using the ggplot2 package in R, presenting the results as volcano plots. The criterion for DEGs was established as FDR < 0.05 and |log2FC| > 1. Additionally, we visualized the variations in DE–BCSCs between LIHC patients and non-tumor controls using the pheatmap and corrplot packages. To investigate the biological functions of DE–BCSCs, we analyzed their roles in three areas: biological processes (BP), cellular components (CC), and molecular functions (MF) as defined by the Gene Ontology (GO) database. Additionally, their associations with KEGG signaling pathways [42,43] were examined using the clusterProfiler tool in R [44]. Following the classification of patients, we constructed a protein–protein interaction (PPI) network to further elucidate the DE–BCSCs, utilizing the STRING database [45].

### 4.3. Designing and Validating Prognostic Models Based on DE–BCSCs

We first assessed the prognostic significance of DE–CSCs in the TCGA cohort through univariate Cox proportional hazards regression analysis, followed by LASSO for genes with *p*-values below 0.05. We created a risk assessment model utilizing the expression data of the selected DE–BCSCs and the coefficients obtained from LASSO. The model was utilized to compute prognostic risk values for patients in the TCGA and ICGC. We proceeded to separate all HCC samples into high-risk and low-risk groups, using the median risk score as the criterion. To analyze the survival variations between the two groups, we used Kaplan–Meier survival analyses with the survival [46] and survminer packages [47]. The ’limma’ package was used to identify DEGs between the high-risk and low-risk groups. The criteria were set at an FDR < 0.05 and |log2FC| > 1. The resulting DEGs were shown through both a volcano plot and a heatmap. We further explored the biological implications of these DEGs by conducting analyses on GO and KEGG pathways, leveraging the clusterProfiler [44] and org.Hs.eg.db [48]. Additionally, GSEA is employed to find enriched pathways (*p*-value ≤ 0.05) [49]. A ridgeline chart presents the top 20 enriched terms. These terms are ranked according to their normalized enrichment scores.

### 4.4. Examining How the Risk Score Can Help in Clinical Diagnosis

To evaluate the clinical efficacy of our risk score more effectively, we constructed time-dependent ROC curves for OS. Concurrently, we determined the area under the ROC curves (AUCs) for 1-year, 3-year, and 5-year intervals across the TCGA and ICGC. These computational endeavors were facilitated using the “timeROC” package in R [25]. OS is the time interval between randomization and death. We also created a risk plot to analyze how the risk score interacts with patients’ prognostic outcomes.

We used a univariate Cox regression analysis, including the risk score, to examine the link between overall survival (OS) and patient characteristics like age, gender, race, tumor stage, and TNM stages. Factors with significant *p*-values (<0.05) in the univariate analysis were included in a multivariate Cox regression analysis. We used forest plots to visually represent the results of both analyses. To implement our findings in clinical practice, we developed a nomogram. This nomogram relies on variables determined through multivariate Cox regression analysis.

We utilized the Oncoplot package for generating a waterfall plot that visualizes gene mutations in the two subgroups. Next, we performed GSEA using the KEGG and HALLMARK gene sets. This analysis aimed to identify differences in pathways and biological processes relevant to the risk-prediction model.

### 4.5. How Prognostic DE–BCSCs Influence Immune Cell Infiltration in Tumors

To assess tumor characteristics, we employed the “ESTIMATE” R package [50] to calculate the stromal score, immune score, ESTIMATE score, and tumor purity for high-risk and low-risk patients. Differences between the two groups were depicted using box plots. We used the CIBERSORT algorithm, well-known deconvolution method, to assess the concentrations of specific immune cell types within tumor specimens [51]. The pheatmap package helped in visualizing the distribution of these immune cell types across individual LIHC patients. We examined the relationships among the 22 immune cell categories in our data set and visualized these findings using the corrplot package. The ggplot2 package visualized the differences in immune infiltration between two risk groups. Moreover, scatter plots were crafted to highlight those gene-immune cell pairs manifesting the strongest positive and negative correlations.

Immune and stromal scores were calculated using the ESTIMATE algorithm. This allowed us to estimate the immune status of each sample in the TCGA-LIHC cohort. We conducted a Pearson correlation analysis to evaluate the relationships among stromal scores, immune scores, and risk scores. Additionally, the influence of the risk-predictive signature on immunotherapeutic outcomes was demonstrated by examining the link between the risk score and identified immune checkpoint genes [36]. Subsequently, we assessed the ratios of these immune cells, highlighting the link between tumor-infiltrating immune cells and the prognostic signature.

### 4.6. Prediction of Chemotherapy Susceptibility

The OncoPredict R package [52] was employed to evaluate drug sensitivity in the TCGA–LIHC cohort. We conducted a comparison of the IC50 values for 10 selected chemotherapeutic agents across high-risk and low-risk groups.

To facilitate this analysis, we compiled a list of potential agents based on relevant literature, publicly available databases, and gene-expression data from patients with LIHC. To validate the therapeutic relevance of the nine signature genes, we analyzed 140 HCC cases from GSE109211 (42 responders vs. 98 non-responders), a data set containing longitudinal therapeutic outcome records.

### 4.7. ScRNA and Cell Communication Analysis of the GSE156625 Data Set

We used Uniform Manifold Approximation and Projection (UMAP) to explore the cellular differences in liver cancer tissues. The UMAP plot displayed a two-dimensional view of the high-dimensional single-cell data, allowing us to visualize important genes that cluster different immune cell populations. We annotated immune cell types using the processed single-cell data from the GSE156625 data set, as described in the reference study.

We analyzed the single-cell data set using Python (version 3.12.7) and LIANA [53] (version 0.1.9, https://saezlab.github.io/liana/, accessed on 12 September 2024) to explore ligand–receptor interactions among cells. We also used the CellPhoneDB (vision 2.1.7) [54] package to analyze the top 20 gene-receptor-ligand pairs, focusing on the significant gene CCL5, which was expressed in our study. The results of cell communication were visualized using a scatter plot, highlighting significant interactions among different cell types. This visualization facilitated the identification of key signaling networks active within the cellular microenvironment.

### 4.8. Statistical Analysis

Without the scRNA data set, R software (version 4.3.1) was used for all further data processing and statistical analyses. The methodologies for the bioinformatics assessments are detailed in the relevant subsections. Statistical significance was denoted as follows: * *p* < 0.05, ** *p* < 0.01, and *** *p* < 0.001. A *p*-value below 0.05 indicates significance.

## 5. Conclusions

This investigation highlights a new prognostic signature for BCSCs in LIHC, comprising nine key genes: ADM, CCL5, CD274, DLGAP5, HOXD9, IGF1, S100A9, SOCS2, and TNFRSF11B. Our analysis reveals significant associations between these genes and OS, indicating that this signature could act as a reliable predictor of patient outcomes. Additionally, the established prognostic model highlights the complex relationship between BCSCs and immune cell infiltration, suggesting that targeting these pathways may provide new opportunities for treatment. These findings underscore the importance of integrating BCSC-related strategies into clinical practice for better patient stratification and personalized treatment in LIHC. Future research should aim to clarify how these BCSCs affect tumor progression and immune dynamics. Ultimately, the goal is to improve therapeutic efficacy and increase patient survival.

## Data Availability

All data used in this study are publicly available from the GDC and the GEO databases.

## Supplementary Materials

The following supporting information can be downloaded at: https://www.mdpi.com/article/10.3390/ijms26072933/s1.

## Abbreviations

The following abbreviations are used in this manuscript:

CSCs	Cancer stem cells
BCSCs	Biomarkers of CSCs
DE–BCSCs	Differentially expressed BCSCs
TCGA	The Cancer Genome Atlas
ICGC	International Cancer Genome Consortium
GEO	Gene Expression Omnibus
scRNA-seq	single-cell RNA sequencing
GO	Gene Ontology
KEGG	Kyoto Encyclopedia of Genes and Genomes
LASSO	Least Absolute Shrinkage and Selection Operator
OS	Overall Survival
TME	Tumor microenvironment
LIHC	Liver hepatocellular carcinoma
HCC	Hepatocellular carcinoma
EMT	Epithelial Mesenchymal Transition
UMAP	Uniform Manifold Approximation and Projection
TNM	Tumor Node Metastasis
TICs	Tumor-Infiltrating Immune Cells
GSEA	Gene Set Enrichment Analysis
LIANA	LIgand-receptor ANalysis frAmework
