# Supplementary material for: Identification of a Cancer Stem Cell-Related Gene Signature in Hepatocellular Carcinoma Based on Single-Cell RNA-Seq and Bulk RNA-Seq Analysis
Source: Int J Mol Sci. 2025 Mar 24;26(7):2933. doi: 10.3390/ijms26072933 (PMC11988464; doi:10.3390/ijms26072933)
Supplement: Supplementary file 1 [file ijms-26-02933-s001.zip › Figure legend S1-S5.pdf]

Figure S1. Univariate Cox regression analysis of DE-BCSCs. Forest plot showing hazard ratios (HR) with 95% confidence intervals (CI) and p-values indicating statistical significance.

Figure S2. Gene expression analysis of nine signature genes in patients treated with sorafenib versus placebo in GSE109211 datasets.

Figure S3. The top 20 ligand-receptor interactions among various immune cells, including B cells, CD4+ T cells, CD8+ T cells, NK cells, and Treg cells.

Figure S4. Flow chart of data collection and analysis. TCGA, The Cancer Genome Atlas; LIHC, Liver Hepatocellular Carcinoma; DE-BCSCs, differentially expressed biomarkers of cancer stem cells; GO, gene ontology; KEGG, Kyoto Encyclopedia of Genes and Genomes; OS, overall survival; LASSO, least absolute shrinkage and selection operator; GSEA, Gene Set Enrichment Analysis.

Figure S5. Comparison of risk scores between the groups stage1_2 and stage3_4
