# Supplementary figures and images for: Identification of a Cancer Stem Cell-Related Gene Signature in Hepatocellular Carcinoma Based on Single-Cell RNA-Seq and Bulk RNA-Seq Analysis
Source: Int J Mol Sci. 2025 Mar 24;26(7):2933. doi: 10.3390/ijms26072933 (PMC11988464; doi:10.3390/ijms26072933)

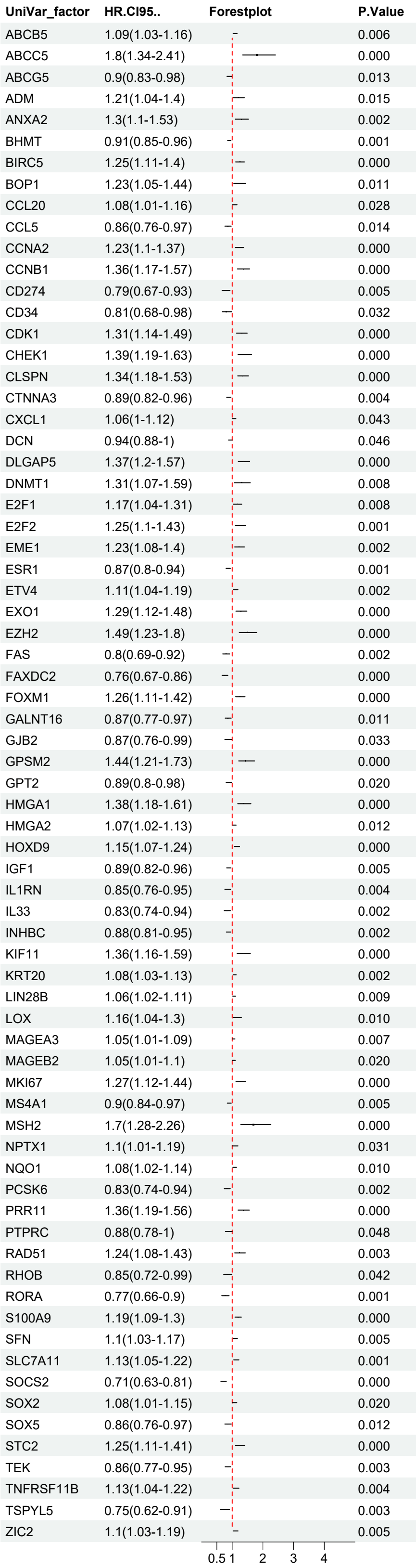

Supplement: Supplementary file 1 [file ijms-26-02933-s001.zip › Figure S1.pdf]

group1 plac sorf

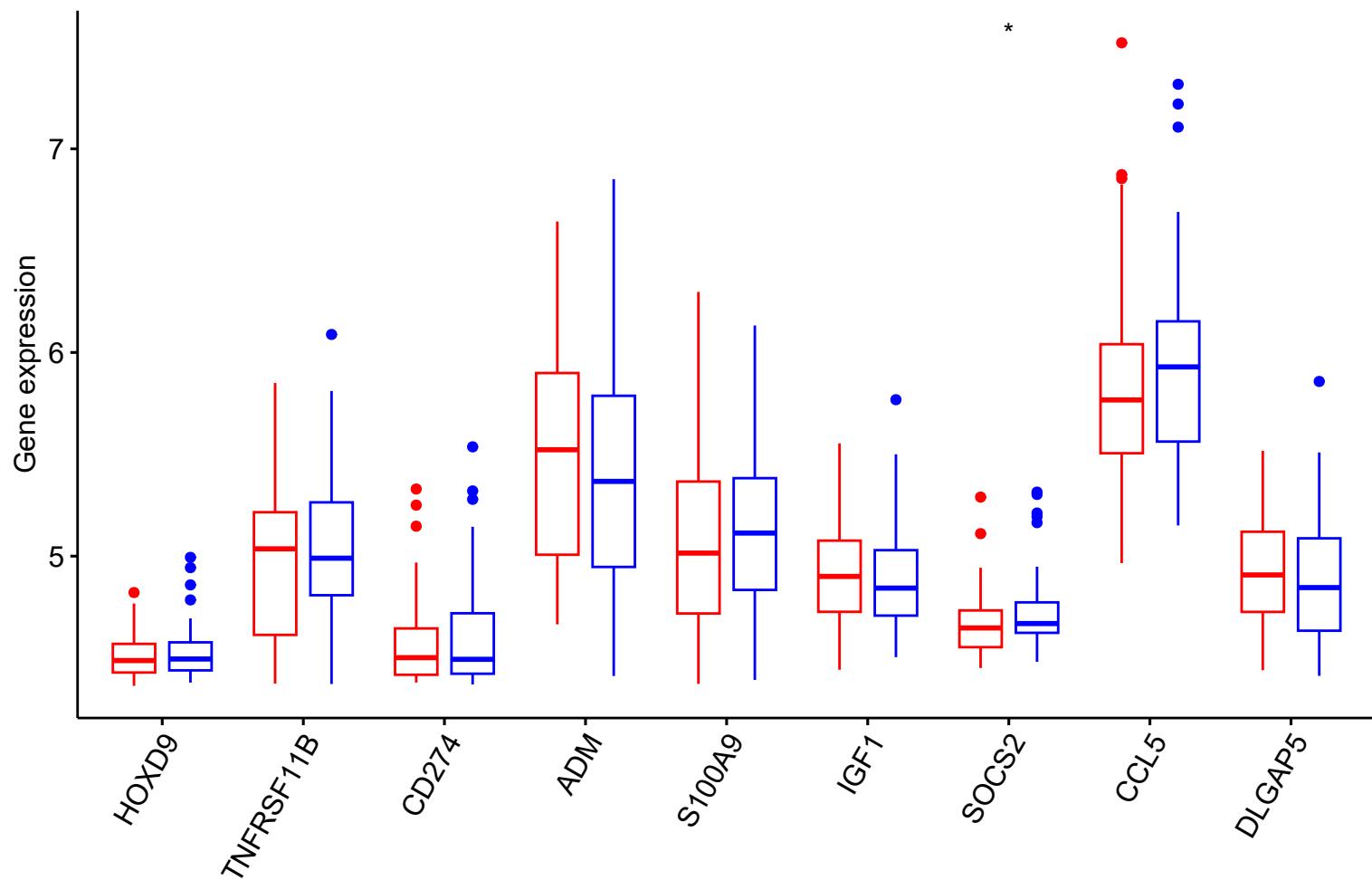

Supplement: Supplementary file 1 [file ijms-26-02933-s001.zip › Figure S2.pdf]

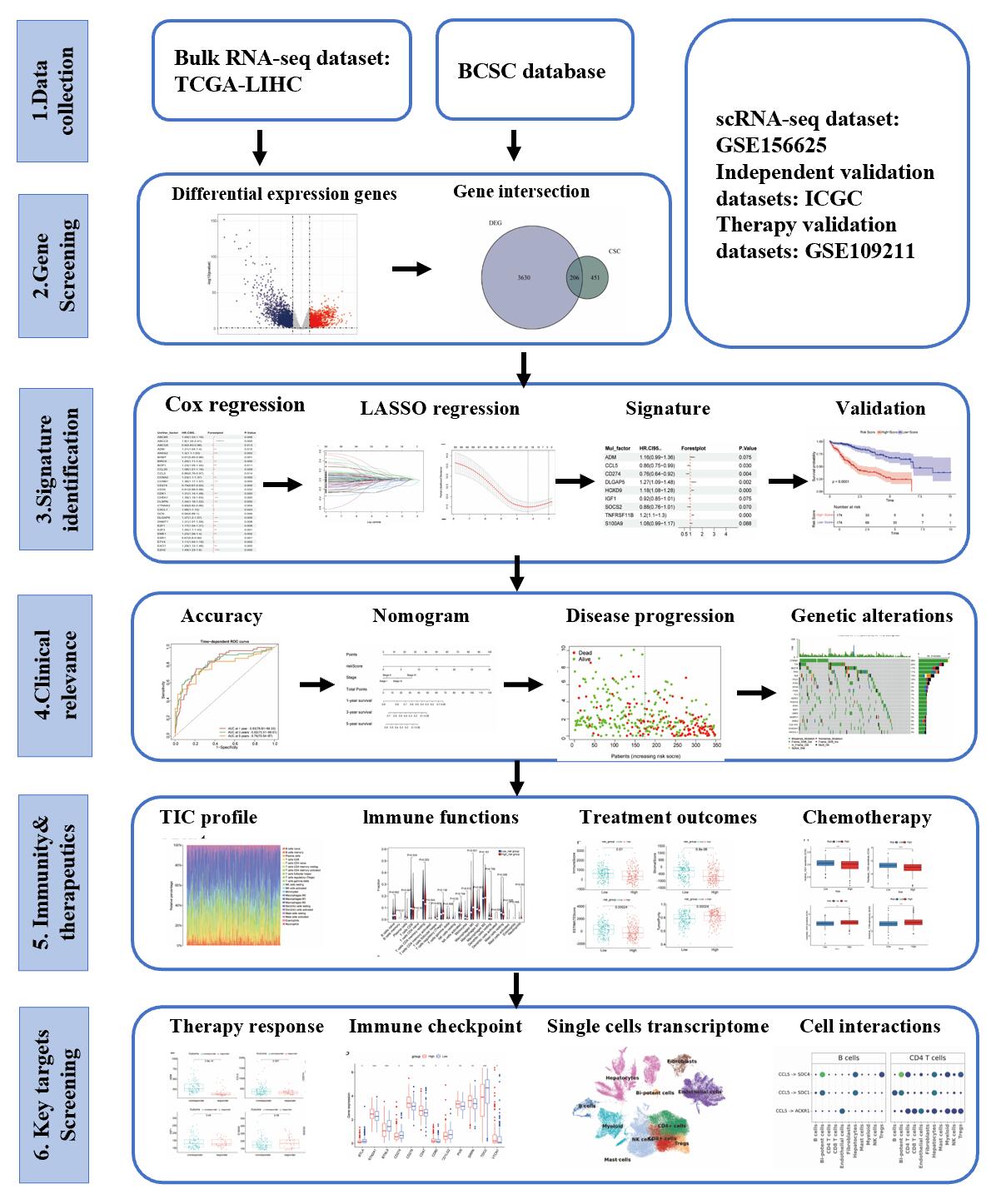

Supplement: Supplementary file 1 [file ijms-26-02933-s001.zip › Figure S4.jpg]

stage1    Stage1\_2    Stage3\_4

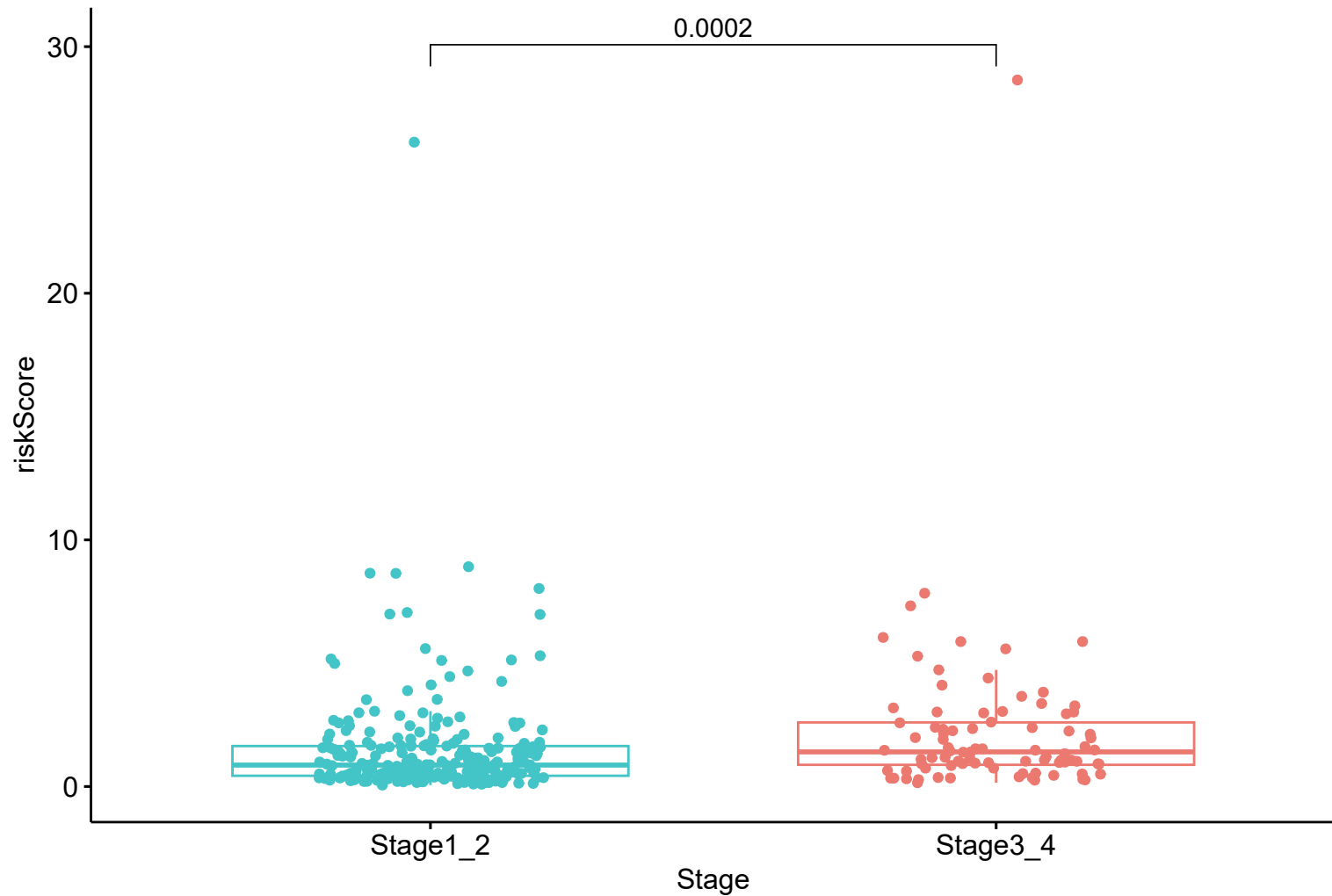

Supplement: Supplementary file 1 [file ijms-26-02933-s001.zip › Figure S5.pdf]
