# Supplementary material for: Identification of a Cancer Stem Cell-Related Gene Signature in Hepatocellular Carcinoma Based on Single-Cell RNA-Seq and Bulk RNA-Seq Analysis
Source: Int J Mol Sci. 2025 Mar 24;26(7):2933. doi: 10.3390/ijms26072933 (PMC11988464; doi:10.3390/ijms26072933)
Supplement: Supplementary file 1 [file Figure S3.pdf › Figure S3.pdf]

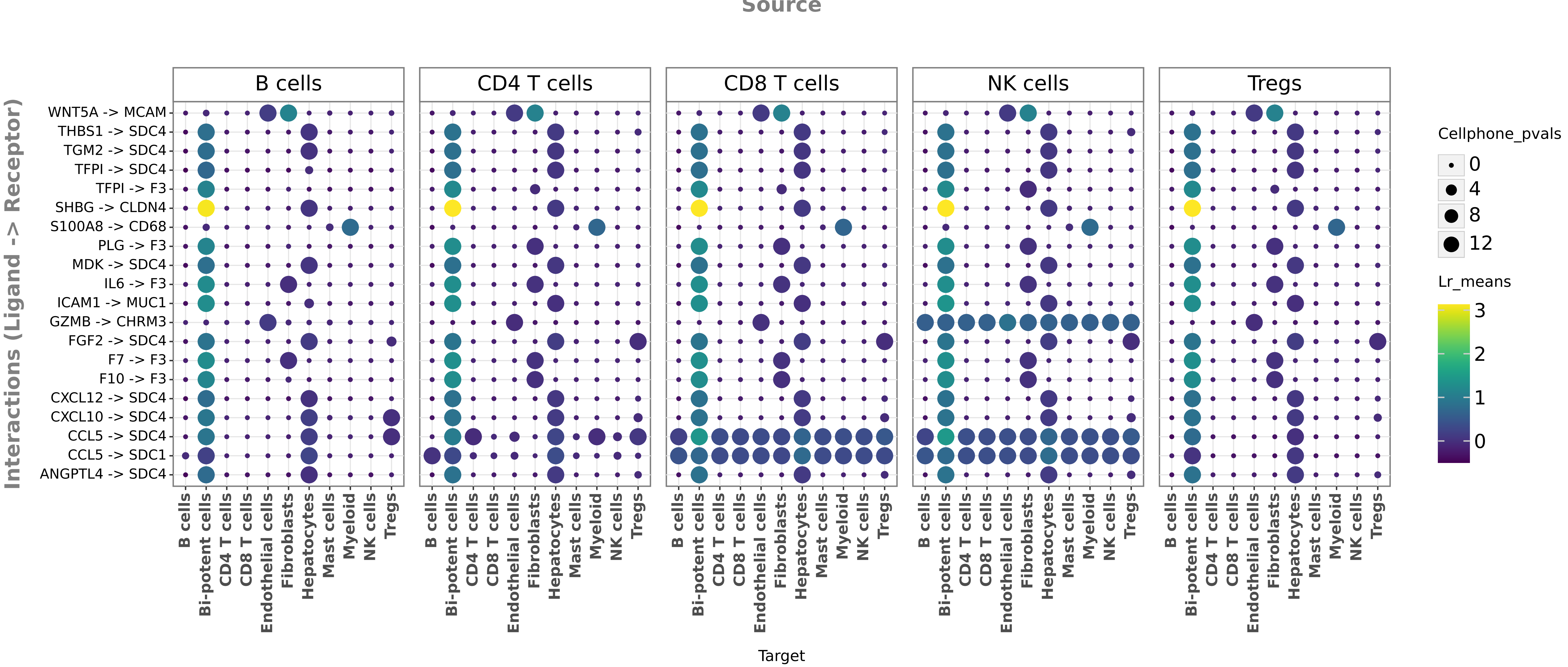
Source
B cells
CD4 T cells
CD8 T cells
NK cells
Tregs
Interactions (Ligand -> Receptor)
WNT5A -> MCAM
THBS1 -> SDC4
TGM2 -> SDC4
TFPI -> SDC4
TFPI -> F3
SHBG -> CLDN4
S100A8 -> CD68
PLG -> F3
MDK -> SDC4
IL6 -> F3
ICAM1 -> MUC1
GZMB -> CHRM3
FGF2 -> SDC4
F7 -> F3
F10 -> F3
CXCL12 -> SDC4
CXCL10 -> SDC4
CCL5 -> SDC4
CCL5 -> SDC1
ANGPTL4 -> SDC4
B cells
Bi-potent cells
CD4 T cells
CD8 T cells
Endothelial cells
Fibroblasts
Hepatocytes
Mast cells
Myeloid
NK cells
Tregs
Target
Cellphone_pvals
0
4
8
12
Lr_means
3
2
1
0
